# Cytological, Phytohormone, and Transcriptome Analyses Provide Insights into Persimmon Fruit Shape Formation (*Diospyros kaki Thunb.*)

**DOI:** 10.3390/ijms25094812

**Published:** 2024-04-28

**Authors:** Huawei Li, Yujing Suo, Hui Li, Peng Sun, Weijuan Han, Jianmin Fu

**Affiliations:** 1 Key Laboratory of Cultivation and Protection for Non-Wood Forest Trees, Ministry of Education, Central South University of Forestry and Technology, No. 498 Shaoshan South Road, Changsha 410004, China; lihuaweicaf@163.com; 2 Research Institute of Non-Timber Forestry, Chinese Academy of Forestry, No. 3 Weiwu Road, Jinshui District, Zhengzhou 450003, China; suoyj@caf.ac.cn (Y.S.); ptsunpeng@caf.ac.cn (P.S.); 3 Research Institute of Forestry Policy and Information, Chinese Academy of Forestry, Xiangshan Road, Haidian District, Beijing 100091, China; lihui09610@163.com

**Keywords:** persimmon, fruit shape, phytohormone, transcriptome

## Abstract

Fruit shape is an important external feature when consumers choose their preferred fruit varieties. Studying persimmon (*Diospyros kaki Thunb.*) fruit shape is beneficial to increasing its commodity value. However, research on persimmon fruit shape is still in the initial stage. In this study, the mechanism of fruit shape formation was studied by cytological observations, phytohormone assays, and transcriptome analysis using the long fruit and flat fruit produced by ‘Yaoxianwuhua’ hermaphroditic flowers. The results showed that stage 2–3 (June 11–June *25*) was the critical period for persimmon fruit shape formation. Persimmon fruit shape is determined by cell number in the transverse direction and cell length in the longitudinal direction. High IAA, GA_4_, ZT, and BR levels may promote long fruit formation by promoting cell elongation in the longitudinal direction, and high GA_3_ and ABA levels may be more conducive to flat fruit formation by increasing the cell number in the transverse direction and inhibiting cell elongation in the longitudinal direction, respectively. Thirty-two DEGs related to phytohormone biosynthesis and signaling pathways and nine DEGs related to cell division and cell expansion may be involved in the persimmon fruit shape formation process. These results provide valuable information for regulatory mechanism research on persimmon fruit formation.

## 1. Introduction

Persimmon (*Diospyros kaki Thunb.*) fruit is very popular in China [1]. There are many sex types of persimmons, such as dioecious, monoecious, andromonoecious, and polygamo-monoecious. Commonly cultivated persimmons are female plants. Horticulturally, fruit shape is an important economic trait. During the sale process, fruit shape is an important consideration for consumers. In production, non-uniformity in fruit shape is not conducive to mechanical harvesting, processing, storage, and transportation. In addition, fruit shape is an important trait selected during breeding [2,3]. The diversity of persimmon fruit shape provides a basis for studying the mechanism of fruit shape regulation, thus facilitating efficient manipulation of fruit shape in breeding.

Fruit shape formation is a complex process involving multiple biological processes, such as cell division, cell expansion, hormone biosynthesis, signal transduction, and key gene expression. In horticultural crops, the fruit shape formation of tomato, cucumber, and peach fruit has been studied intensively [2,4,5]. In tomato, the application of exogenous auxin and gibberellin can produce elongated fruit, whereas the application of gibberellin inhibitor paclobutrazol results in flatter fruits [6,7,8]. With the development of sequencing technology, several genes thought to control the fruit shape of tomato were identified [9], such as locule number (*LC*) and fascinated (*FAS*), which affect fruit shape by regulating locule number, *SUN*, which encodes for a protein that positively regulates fruit elongation [10], and *OVATE*, which encodes a negative regulator of growth that reduces fruit length [11]. In cucumber, ABA, mediated by *CsTRM5*, can change the fruit shape by regulating cell division and expansion [12]. In peach, auxin plays an important role in regulating fruit shape [13].

Current research on persimmon fruit shape is mainly descriptive, and there are relatively few in-depth studies [14]. Here, the flat and long fruit of ‘Yaoxianwuhua’, which were in the critical morphological periods of fruit shape formation, were used for phytohormone assays and transcriptome analysis to determine the regulatory roles of phytohormones and candidate genes in fruit shape formation. This study provides valuable information for further exploring the mechanism of persimmon fruit shape formation.

## 2. Results

### 2.1. Morphological Comparison of Fruit

**Figure 1:**
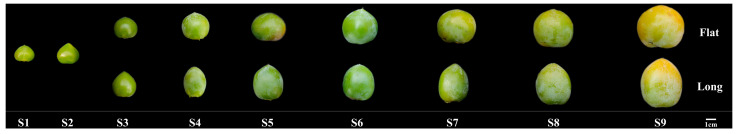
Morphological changes of ‘Yaoxianwuhua’ fruit.

**Figure 2:**
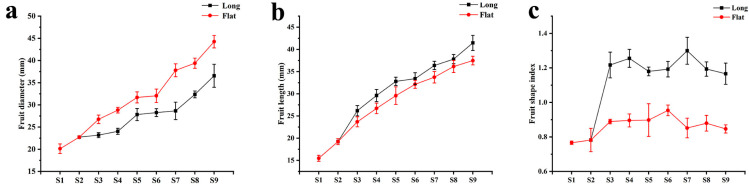
Changes in FD, FL, and FSI during fruit development. (**a**) Change in FD. (**b**) Change in FL. (**c**) Change in FSI.

The ‘Yaoxianwuhua’ persimmon is polygamo-monoecious with male flowers, female flowers, and hermaphroditic flowers. It is interesting that the hermaphroditic flowers can produce flat fruit and long fruit. In order to study the reasons for the different fruit shape formation, we observed the phenotypes of fruit throughout the development period. At stage 1–2 (May 29–June 11), only one relatively flat fruit type was produced by hermaphroditic flowers. At stage 3 (June 25), the fruit diameter (FD), length (FL), and shape index (FSI: length-by-diameter ratio) began to differentiate. Some fruit developed into long fruit with larger FL and FSI, and some fruit developed into flat fruit with smaller FD and smaller FSI. At stage 4–9 (July 8–September 15), the FD and FL increased steadily, but the FSI fluctuated slightly and the fruit shape did not change (Figure 1 and Figure 2). Therefore, stage 2–3 (June 11–June 25) was crucial to persimmon fruit shape formation.

**Figure 3:**
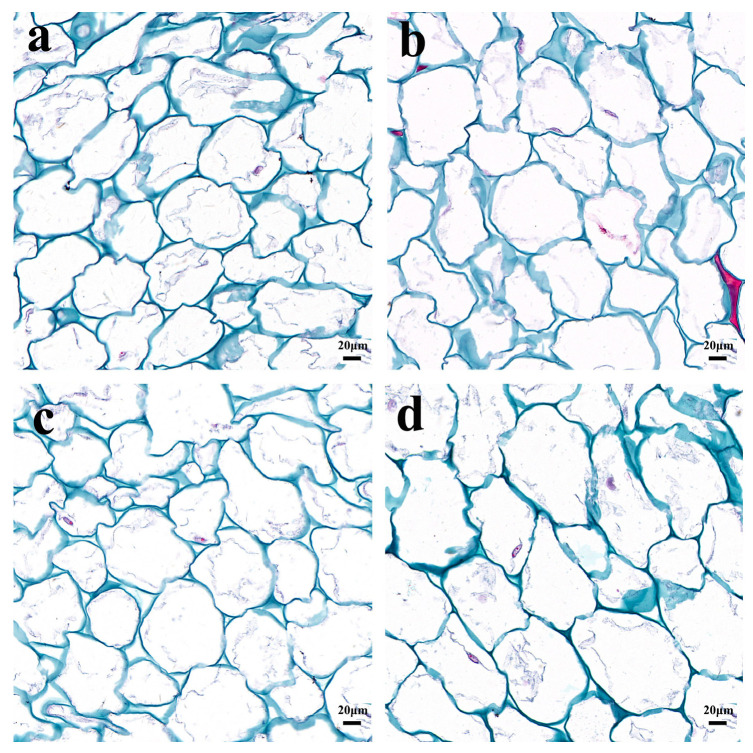
Histological observations of fruit. (**a**) Transverse section of flat fruit. (**b**) Longitudinal section of flat fruit. (**c**) Transverse section of long fruit. (**d**) Longitudinal section of long fruit.

**Figure 4:**
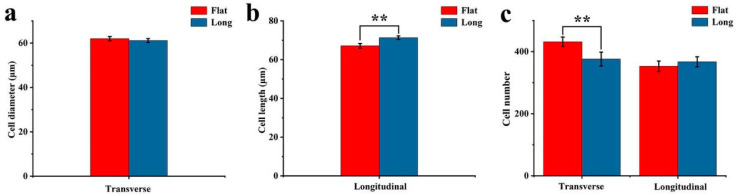
Statistics on fruit cell diameter, length, and number. (**a**) Cell diameter in the transverse direction. (**b**) Cell length in the longitudinal direction. (**c**) Cell number in the transverse direction and in the longitudinal direction. **: *p <* 0.01.

In order to observe the morphological differences of fruit cells, the fruit tissues were sliced (Figure 3). In the transverse direction, there was no difference in cell diameter between flat fruit and long fruit (Figure 4a), while the cell number of flat fruit was significantly more than that of long fruit (Figure 4c), indicating that cell number was responsible for the difference in diameter between long fruit and flat fruit. In the longitudinal direction, there was no difference in cell number between flat fruit and long fruit (Figure 4b), while the cell length of long fruit was significantly larger than that of flat fruit (Figure 4c), indicating that cell length was responsible for the difference in length between long fruit and flat fruit. In summary, persimmon fruit shape was determined by cell number in the transverse direction and cell length in the longitudinal direction.

### 2.2. Phytohormone Content in Flat and Long Fruits

**Figure 5:**
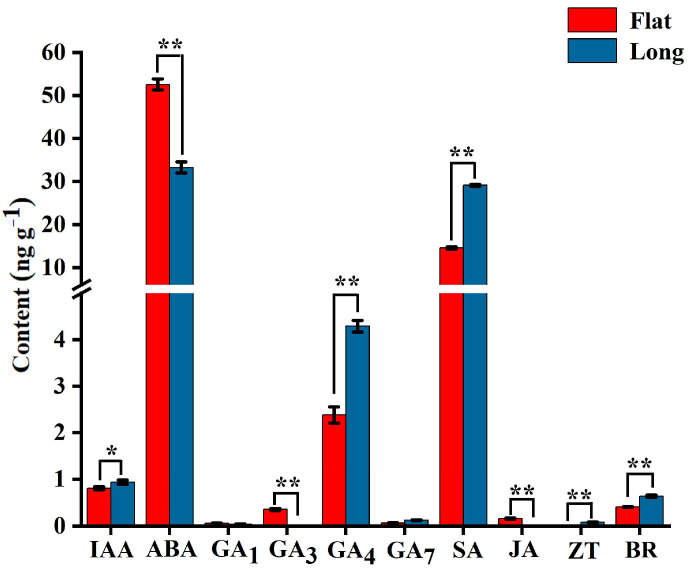
Phytohormone levels in flat and long fruit. *: *p <* 0.05; **: *p <* 0.01.

To establish the effects of endogenous phytohormones on fruit shape, we measured auxin (IAA), abscisic acid (ABA), gibberellin (GA_1_, GA_3_, GA_4_ and GA_7_), salicylic acid (SA), jasmonic acid (JA), zeatin (ZT), and brassinosteroid (BR) levels in flat and long fruit at stage 3. The IAA, GA_4_, SA, ZT, and BR levels in long fruit were markedly higher than those in flat fruit, while the ABA, GA_3_, and JA levels in flat fruit were significantly higher than those in long fruit (Figure 5). These results showed that phytohormones play an important role in persimmon fruit shape formation.

### 2.3. Transcriptome

To identify the mRNA expression profiles in long fruit and flat fruit, six cDNA libraries at stage 3 (F1, F2, and F3 for flat fruit and L1, L2, and L3 for long fruit) were constructed and sequenced on the NovaSeq 6000 platform. A total of 22.28, 21.44, 25.16, 22.34, 29.32, and 22.62 Mb clean reads were obtained, respectively (Table S1). Above 85.50% of the reads mapped to the reference *D. kaki* genome (Table S2).

**Figure 6:**
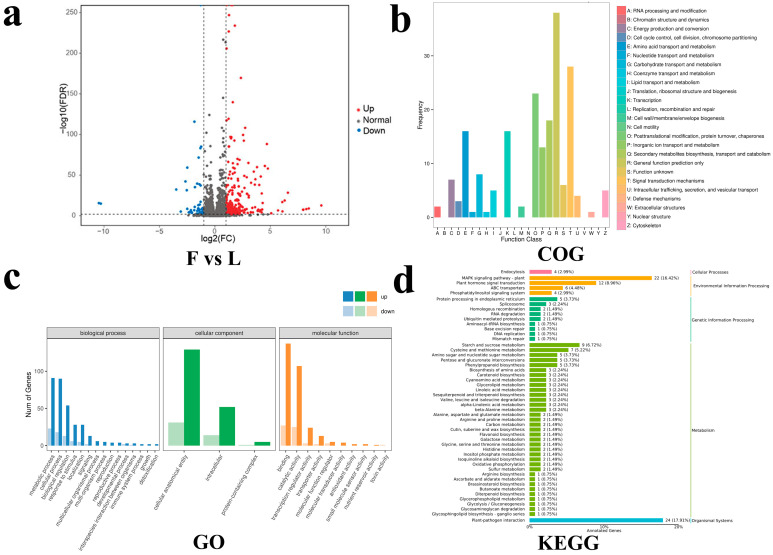
Number and functional classification of DEGs. (**a**) The volcano plot shows the number of DEGs in F vs. L. (**b**) COG-annotated classification statistical map of DEGs. (**c**) GO-annotated classification statistical map of DEGs. (**d**) KEGG-annotated classification statistical map of DEGs.

The differentially expressed genes (DEGs) were compared using DESeq2 software (v.1.20.0). A total of 380 DEGs were identified between flat and long fruits. Compared with flat fruit, 311 genes were upregulated and 69 were downregulated in long fruit (Figure 6a). The functional classification and statistics of the DEGs showed that the most abundant COG categories were signal transduction mechanisms and carbohydrate transport and metabolism (Figure 6b). The most abundant GO categories were binding and cellular anatomical entity (Figure 6c). The most abundant KEGG pathways were plant–pathogen interaction, MAPK signaling pathway-plant and plant hormone signal transduction (Figure 6d). 

### 2.4. DEGs Related to Phytohormones

**Figure 7:**
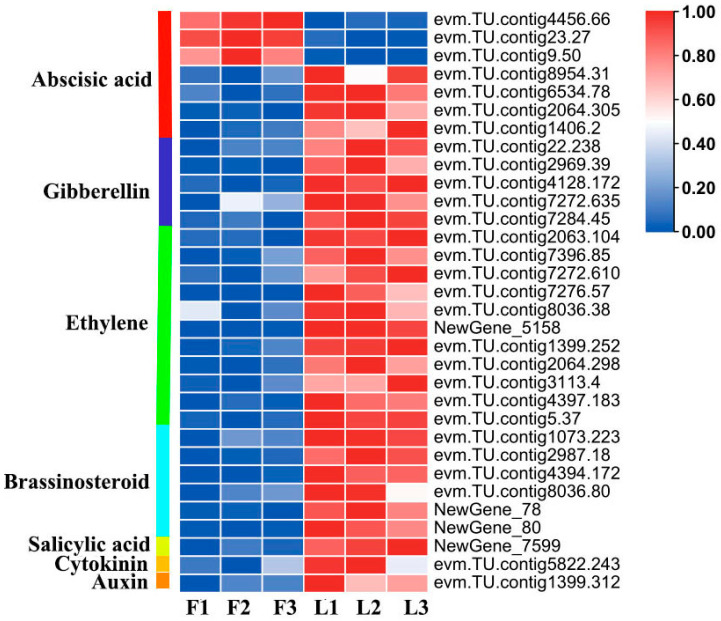
Heat map of DEGs related to phytohormone biosynthesis and signal transduction pathways. The original expression values of the DEGs in FPKM (fragments per kilobase per million) were normalized by Z-score.

The combined analysis of phytohormones and the transcriptome identified 32 DEGs related to phytohormone biosynthesis and signal transduction pathways. Among them, 29 DEGs were upregulated in long fruit and three DEGs were upregulated in flat fruit (Figure 7; Table S3). 

In the abscisic acid signal transduction pathway, *PP2C* (*evm.TU.contig23.27* and *evm.TU.contig9.50*) and *CYP707A1* (*evm.TU.contig4456.66*) were downregulated in long fruit, while *CYP707A4* (*evm.TU.contig8954.31*), *CSBP* (*evm.TU.contig6534.78*), *FRAA2* (*evm.TU.contig2064.305*), and *ACR8* (*evm.TU.contig1406.2*) were downregulated in long fruit. 

In the gibberellin signal transduction pathway, *CYP714C2* (*evm.TU.contig22.238*), *LBD41* (*evm.TU.contig2969.39*), *CIGR1* (*evm.TU.contig4128.172*), *EFM* (*evm.TU.contig7272.635*), and *SCL13* (*evm.TU.contig7284.45*) were upregulated in long fruit. 

In the brassinosteroid signal transduction pathway, *CYP90C1* (*evm.TU.contig1073.223*), *RKL1* (*evm.TU.contig2987.18*), *PHI-1* (*evm.TU.contig4394.172*), *At4g25390* (*evm.TU.contig8036.80*) and *XTH23* (*NewGene_78* and *NewGene_80*) were upregulated in long fruit. 

In the salicylic acid biosynthesis pathway, *2ODD19* (*NewGene_7599*) was upregulated in long fruit. 

In the cytokinin biosynthesis pathway, *CKX7* (*evm.TU.contig5822.243*) was upregulated in long fruit. 

In the auxin signal transduction pathway, *SAUR36* (*evm.TU.contig1399.312*) was upregulated in long fruit.

Ethylene plays an important role in plant growth and development. In this study, the DEGs associated with ethylene biosynthesis and signal transduction pathways were also identified. Eleven genes, including *ETR2* (*evm.TU.contig2063.104*), *MKK5* (*evm.TU.contig7396.85*), *EBF1* (*evm.TU.contig7272.610*), *ERF5* (*evm.TU.contig1399.252* and *evm.TU.contig5.37*), *ERF012* (*evm.TU.contig3113.4*), *ERF014* (*evm.TU.contig4397.183*), *ERF017* (*evm.TU.contig7276.57*), *ERF106* (*evm.TU.contig8036.38*), *ACO1* (*NewGene_5158*), and *RAP2–3* (*evm.TU.contig2064.298*), were upregulated in long fruit.

### 2.5. DEGs Related to Cell Division and Cell Expansion

**Figure 8:**
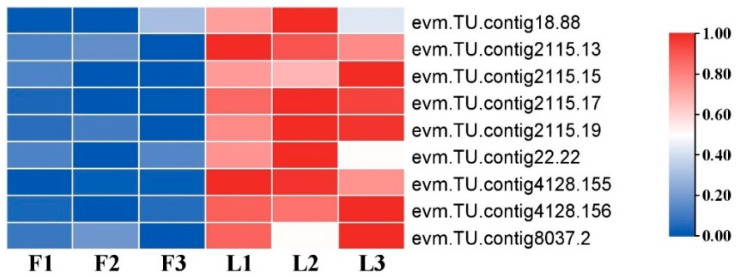
Heat map of DEGs related to cell division. The original expression values of the DEGs in FPKM were normalized by Z-score.

Plant growth and development are closely related to cell division and cell expansion. Nine DEGs related to cell division, including *JGB* (evm.*TU.contig18.88*), *AATP1* (*evm.TU.contig2115.13*, *evm.TU.contig2115.15*, *evm.TU.contig2115.17* and *evm.TU.contig2115.19*), UBP12 (*evm.TU.contig22.22*), *KRP1* (*evm.TU.contig4128.155* and *evm.TU.contig4128.156*), and *At2g46620* (*evm.TU.contig8037.2*), were identified in flat and long fruit. They were upregulated in long fruit (Figure 8; Table S4).

### 2.6. DEGs Validation by RT-qPCR

**Figure 9:**
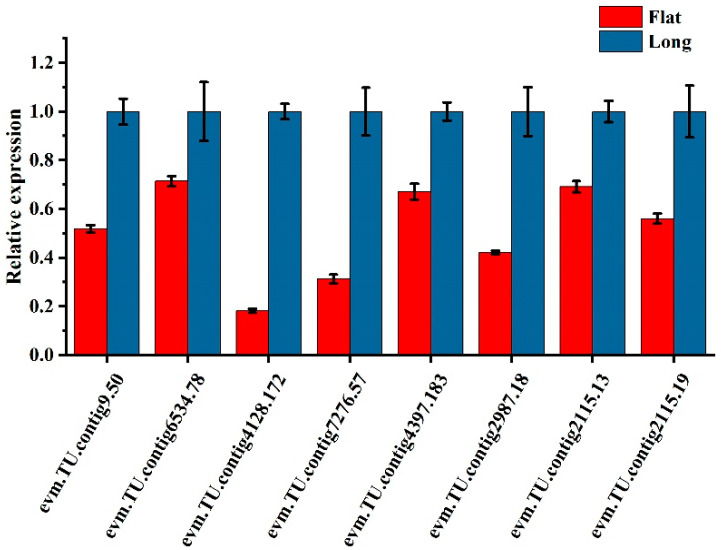
DEG validation by RT-qPCR.

Eight DEGs were selected for RT-qPCR analysis to assess transcriptome data accuracy. The expression patterns of DEGs were consistent with the RNA-seq results. Thus, our sequencing data were reliable (Figure 9).

## 3. Discussion

There is abundant variation in fruit shape among persimmon cultivars [14]. The most common persimmon cultivar is hexaploid (2n = 6x = 90) with a complex genetic background, which results in a rather complex regulatory mechanism for fruit shape formation [15]. The ‘Yaoxianwuhua’ persimmon is polygamo-monoecious with male flowers, female flowers, and hermaphroditic flowers, and the hermaphroditic flowers can produce flat fruit and long fruits. Using its hermaphroditic flower fruit as research materials to study the mechanism of persimmon fruit shape formation can effectively eliminate the interference of inconsistent genetic backgrounds of experimental materials and obtain more reliable results.

Fruit shape formation is inseparable from cell division and cell expansion [16,17]. Corresponding to cell division and cell expansion, fruit cell number and size play crucial roles in fruit shape formation. Some studies have provided evidence that differences in shape are mainly determined by cell number and size, such as melon [18], sweet cherry [19], peach [20], cucumber [21], and pear [22]. In this study, the cell length of long fruit was larger than that of flat fruit in the longitudinal direction and the cell number of flat fruit was more than that of long fruit in the transverse direction, which suggested that persimmon fruit shape is determined by cell number in the transverse direction and cell length in the longitudinal direction. The differences in cell number and length were caused by cell division and cell expansion, respectively. The transverse division of fruit cells is conducive to the increase in fruit diameter to promote flat fruit formation. The longitudinal elongation of fruit cells is conducive to the increase in fruit length to promote long fruit formation.

Cell division and cell expansion are inseparable from gene expression. Several genes involved in cell division related to fruit shape formation were found in some studies. For example, the vertical development of apple fruit was affected by *ANT1* and *ANT2*, which are related to cell division [23]. In crops, *WTG1* influences grain size and shape by regulating cell expansion [24]. In this study, nine DEGs related to cell division and cell expansion were identified between flat and long fruit, which may be involved in regulation of the cell number and elongation during persimmon fruit shape formation. 

Phytohormones usually directly or indirectly regulate fruit shape formation. Auxin, cytokinin, gibberellin, and brassinosteroid have been shown to control fruit shape in plants. Application of auxin can increase the number of cells at the proximal end along the longitudinal axis to promote the elongation of tomato fruit [6]. Several members of the *Auxin Response Factor* (*ARF*) family, including *ARF2*, *ARF7*, and *ARF10*, have been also shown to impact tomato fruit shape in distinct ways [25,26,27]. The content of IAA in round peaches was significantly higher than that in flat peaches, and four genes related to the auxin signaling pathway were identified to be involved in regulating flat fruit shape formation [13]. Gibberellin is a hormone well correlated with cell division. A recent study demonstrated that the *SlymiR159-SlGAMYB2* pathway is involved in regulation of fruit shape formation by modulating GA biosynthesis in tomato [7]. In pear, GAs are involved in the regulation fruit shape [28]. BR regulates many processes, including fruit development. The enhancement of BR signaling transduction can increase grain length and decrease grain thickness and width by regulating cell division in rice [29,30]. Several studies on Arabidopsis and rice also demonstrated that interaction of the BR and GA signaling pathways can regulate cell elongation and plant development [31,32,33,34,35]. External application of auxin, GA_4_, and BR can induce the transverse arrangement of microtubules in cells and thereby promote cell elongation in Arabidopsis [36]. The above studies have proven that IAA, GAs, and BR not only promote cell division, but also promote cell elongation, and their functions may be different in different plants. In this study, the IAA, GA_4_, and BR levels in long fruit were higher than those in flat fruit, indicating high IAA, GA_4_, and BR levels may contribute to long persimmon fruit formation by promoting cell elongation in the longitudinal direction. Cell elongation during this process may be related to the transverse arrangement of microtubules in cell induced by IAA, GA_4_, and BR. Meanwhile, the GA_3_ level in flat fruit was higher than that in long fruit, indicating that high GA_3_ levels may contribute to flat persimmon fruit formation by increasing cell number in the transverse direction. The different regulatory patterns of GA_3_ and GA_4_ in persimmon fruit shape formation are worthy of further study. In cucumber, application of the *CKX* inhibitor thidiazuron partially complemented the short fruit phenotype and the treatment had a stronger effect in sf2 mutant (its fruit length is reduced by 50% compared to wild-type) than in wild-type. This was consistent with the effect of *SF2* on promoting cell division by regulating the cytokinin content [37]. In addition to regulating cell division, cytokinin also regulates cell expansion during plant growth and development. In kiwifruit, cytokinin can promote fruit cell expansion [38]. In rice, cytokinin stimulates lateral root elongation by promoting cell elongation [39]. In this study, the ZT levels in long fruit were higher than those in flat fruit, indicating that high ZT levels may contribute to long persimmon fruit formation by promoting cell elongation in the longitudinal direction. In the transcriptome data, 14 DEGs related to auxin, cytokinin, gibberellin, and brassinosteroid biosynthesis and signaling transduction pathways were highly expressed in long fruit. This result indicated that these genes play important roles in persimmon fruit shape formation.

During fruit development, the function of abscisic acid is often opposite to that of auxin and gibberellin. For example, the ABA content in strawberry fruit was higher on flower opening day. With the increase in GA and IAA contents, the ABA content begins to decline [35]. In this study, the ABA level in flat fruit was higher than that in long fruit, while the GA_4_ and IAA levels were lower than those in long fruit, indicating that high ABA may be more conducive to flat persimmon fruit formation by inhibiting cell elongation on longitudinal direction.

Evidence that ethylene plays an important role in controlling fruit shape is that high expression of *EIN3-binding F-box protein2-like* (*SlEBF2-like*), a negative regulatory factor of the ethylene signaling pathway, leads to elongated fruit with increased fruit length and decreased fruit diameter in tomato [40,41]. In this study, the *EBF1* gene was highly expressed in long fruit. In addition, ten genes related to ethylene biosynthesis and signal transduction, including *ETR2*, *MKK5*, *ERF5*, *ERF012*, *ERF014*, *ERF017*, *ERF106*, *ACO1*, and *RAP2–3*, were identified to be highly expressed in long fruit. These results suggest that ethylene may be involved in the regulation of persimmon fruit shape formation.

Salicylic acid and jasmonic acid are involved in many biological processes, such as stress, disease defense, root growth, and development [42,43]. At present, there are no reports on the regulation of SA and JA in fruit shape formation. In this study, the SA content in long fruit was high than that in flat fruit, while the JA content in flat fruit was higher than that in long fruit. The roles of SA and JA in persimmon fruit shape formation deserve further study.

## 4. Conclusions

In conclusion, cell number and length affect persimmon fruit shape by regulating the diameter and length, respectively. The differences in cell number and length were caused by cell division and cell expansion. The transverse division of fruit cells is conducive to the increase in fruit diameter to promote flat fruit formation. The longitudinal elongation of fruit cells is conducive to the increase in fruit length to promote long fruit formation. High IAA, GA_4_, ZT, and BR levels may promote long fruit formation by promoting cell elongation in the longitudinal direction, and high GA_3_ and ABA levels may be more conducive to flat fruit formation by increasing the cell number in the transverse direction and inhibiting cell elongation in the longitudinal direction, respectively. Ethylene may also be involved in persimmon fruit shape formation. The roles of SA and JA in persimmon fruit shape formation deserve further study. Thirty-two genes related to phytohormone biosynthesis and signaling pathways and nine genes related to cell division and cell expansion may be involved in the persimmon fruit shape formation process. This study lays an empirical foundation for ongoing investigations of persimmon fruit shape formation.

## 5. Materials and Methods

### 5.1. Plant Material

The ‘Yaoxianwuhua’ persimmon (*Diospyros kaki*) were ten-year-old trees in Yuanyang County, Henan Province, China (34°55′18^2^~34°56′27^2^ N, 113°46′14^2^~113°47′35^2^ E). After 8 years of field observation, all fruit development stages had stable horticultural characteristics. Between 29 May 2022 and 15 September 2022, flat and long fruit were randomly collected every 14 days. Some fruit samples were fixed in FAA. The volume ratio of formalin, glacial acetic acid, and 50% alcohol in FAA was 8:58:7. Other fruit samples were immediately wrapped in tinfoil, frozen in liquid nitrogen, and stored at −80 °C for subsequent phytohormone testing and RNA extraction.

### 5.2. Paraffin Section

The fruit samples taken out of the FAA were dehydrated by ethanol and then embedded in paraffin. The samples were sectioned using a Leica RM2265 microtome (Leica Microsystems, Nussloch, Germany). The deparaffinized and rehydrated sections were stained with toluidine blue for 30 min. Then, the section was dried on a microslide and mounted with a cover slip. Finally, the sample was observed and pictures were taken using a light microscope (Olympus, Tokyo, Japan).

The diameter and length of fruit cells in the given images were calculated using ImageJ software (v.1.8.0). Cell diameter is the length in the transverse direction. Cell length is the length in the longitudinal direction. The cell number in the transverse direction = fruit diameter/fruit cell diameter. The cell number in the longitudinal direction = fruit length/fruit cell length. Six samples were counted for each development stage.

### 5.3. Phytohormone Assay

Frozen fruit sample tissues were ground in a mortar with liquid nitrogen. About 50 mg FW of fruit samples were weighed and packed into 2 mL centrifuge tube. 

The IAA, ABA, GA_1_, GA_3_, GA_4_, GA_7_, SA, JA, and ZT assays: add internal standards, 7.5 μL d_5_-IAA (2 ng/μL), 40 μL d_6_-ABA (0.25 ng/μL), 10 μL d_2_-GA_1_ (2 ng/μL), 10 μL d_2_-GA_3_ (2 ng/μL), 10 μL d_2_-GA_4_ (2 ng/μL), 10 μL d_2_-GA_7_ (2 ng/μL), 10 μL d_6_-SA (2 ng/μL), 5 μL H_2_-JA (0.25 ng/μL), and 5 μL d_5_-ZT (0.25 ng/μL), into a centrifuge tube and shake well. Then, add 0.5 mL of the extract (C_3_H_8_O:H_2_O:HCl = 2:1:0.002) into the centrifuge tube. Shake well at 100 rpm for 30 min at 4 °C. Next, take out the centrifuge tube and add 1 mL CH_2_Cl, and centrifuge at 12,000 rpm for 5 min at 4 °C. Draw the solvent from the lower phase into a new centrifuge tube and dry it using a nitrogen evaporator with nitrogen flow. Then, add 0.2 mL CH_3_OH to the new centrifuge tube and centrifuge for 5 min. Absorb the supernatant and put it into a small bottle for HPLC-MS/MS. 

The BR contents assay: add internal standard, 5 μL [^2^H_3_]BL (0.25 ng/μL), into centrifuge tube and shake well. Then, add 1 mL CH₃OH of pre-cooled at 4 °C and extract at 4 °C for 2 h. Extract the supernatant by centrifugation at 10,000 rpm for 5 min at 4 °C. Then, add 0.5 mL CH₃OH to wash the residue and centrifuge for 2 h. Combine the two collected supernatants. After the supernatant is dried using a nitrogen evaporator, add 0.2 mL CH₃OH to dissolve it. Finally, filter the solution with a 0.22 μm filter membrane and put it into a small bottle for HPLC-MS/MS.

Samples were injected into an Agilent SB-C18 column and separated at a flow rate of 0.8 mL/min with the following mobile phases. The injection volume was 5.0 μL. Data collection and processing was carried out in AB SCIEX Analyst v.1.7

### 5.4. Transcriptome

Total RNA for the sequencing libraries was extracted using TRIzol Total RNA Isolation Kit (Sangon, Shanghai, China). The RNA integrity was tested by the Bioanalyzer 2100. The libraries were sequenced on the NovaSeq 6000 platform (Illumina, San Diego, CA, USA), and 150 bp paired-end readings were generated. Using the persimmon genome as a reference, HISAT2 software (v.2.2.1) was used to match the filtered reads [15,44]. The new transcripts were predicted using StringTie software (v.2.1.1) [45]. The number of reads mapped to each gene were calculated using FeatureCounts software (v.2.0.1) [46]. FPKM was used to represent gene expression. The differential mRNA expression (DEGs) (fold change ≥ 2; FDR < 0.01) was detected by DEseq2.

### 5.5. Quantitative RT-PCR

Total RNA was reverse-transcribed into cDNA using the TRUE-script 1st-Strand cDNA Synthesis Kit (Kemix, Beijing, China). Quantification of DEG expression was detected with TB Green™ Premix Ex Taq™ II (Tli RNaseH Plus) (Takara, Dalian, China). The reaction conditions were: 30 s at 95 °C, 40 cycles of 3 s at 95 °C, and 30 s at 60 °C. Three technical replicates were analyzed. The reference gene was *GAPDH* [47]. The relative expression was calculated using the 2^−ΔΔCt^ method. The DEG primers are listed in Table S5.

## Data Availability

The transcriptome sequencing raw data were deposited in the National Center for Biotechnology Information Sequence Read Archive (NCBI SRA) under the Bioproject ID PRJNA1094889.

## Supplementary Materials

The following supporting information can be downloaded at: https://www.mdpi.com/article/10.3390/ijms25094812/s1.
